# Diagnostic and Predictive Values of Inflammatory Factors in Pathology and Survival of Patients Undergoing Total Cystectomy

**DOI:** 10.1155/2020/9234067

**Published:** 2020-09-24

**Authors:** Xingxing Tang, Yudong Cao, Jia Liu, Shuo Wang, Yong Yang, Peng Du

**Affiliations:** Key Laboratory of Carcinogenesis and Translational Research (Ministry of Education), Department of Urology, Peking University Cancer Hospital & Institute, No. 52 Fucheng Road, Haidian District, Beijing, China

## Abstract

**Background:**

Inflammation and tumorigenesis are related. We conducted this study to evaluate whether inflammatory factors (IFs) have a diagnostic value for pathology and a predictive value for survival and recurrence in bladder cancer patients undergoing total cystectomy.

**Methods:**

The patients who were diagnosed with bladder cancer and underwent total cystectomy in our center from 2014 to 2020 were enrolled. The values of neutrophil to lymphocyte ratio (NLR), derived neutrophil to lymphocyte ratio (dNLR), platelet to lymphocyte ratio (PLR), lymphocyte to monocyte ratio (LMR), and systemic immune-inflammation index (SII) were calculated by blood routine test results before operation. The AUC of ROC was calculated to judge the diagnostic value of the IFs in pathology and their corresponding cut-off values. For overall survival (OS) and recurrence-free survival (RFS), the above IFs were grouped according to the cut-off value. The differences between different groups were analyzed by the Kaplan-Meier curves, and the predictive value of these IFs was determined by the Cox proportional hazards regression model.

**Results:**

A total of 79 patients were enrolled. All IFs had no diagnostic value for the pathological grade, tumor T stage, and systemic metastasis. Only NLR (AUC = 0.706, cut‐off value = 3.12, sensitivity = 75.00%, specificity = 70.00%, *P* = 0.014), dNLR (AUC = 0.700, cut‐off value = 2.49, sensitivity = 66.67%, specificity = 76.67%, *P* = 0.015), and SII (AUC = 0.704, cut‐off value = 463.56, sensitivity = 100.00%, specificity = 40.00%, *P* = 0.004) had a diagnostic value for lymph node metastasis. The median follow-up time was 31 months, and there was no significant difference in OS between the two groups for all IFs. For RFS, Kaplan-Meier suggested PLR might be predictive when the cut-off value was 266.70 (*P* = 0.044), but the subsequent Cox proportional hazards regression analysis showed that all IFs had no predictive value for OS and RFS.

**Conclusions:**

We found that in patients undergoing total cystectomy preoperative NLR, dNLR and SII had a diagnostic value for lymph node metastasis, while all these five IFs had no predictive value for OS and RFS. However, this conclusion needs to be further verified by large-scale studies in the future.

## 1. Introduction

At present, many studies have confirmed that there is a certain link between inflammation and the occurrence and development of tumors [1]. A series of inflammatory factors (IFs) derived from peripheral blood test results have been confirmed to have a certain diagnostic value in a variety of tumors, mainly including neutrophil to lymphocyte ratio (NLR), derived neutrophil to lymphocyte ratio (dNLR), platelet to lymphocyte ratio (PLR), lymphocyte to monocyte ratio (LMR), and systemic immune-inflammation index (SII) [2, 3]. Because the peripheral blood test is a routine examination for patients admitted to hospital, it is very convenient to obtain the aforementioned IFs, which can be directly calculated from the results of the routine blood test without increasing the expenditure and pain of patients. Bladder urothelial carcinoma (hereinafter referred to as bladder cancer) is a common tumor in urology; if the disease invades the muscle or is widely distributed in the bladder and cannot be completely removed by transurethral resection of bladder tumor (TURBT), patients usually need to undergo total cystectomy [4]. Preoperative evaluation of the depth of tumor invasion, lymph node metastasis, and systemic metastasis in these patients is helpful for clinicians to draw a more favorable treatment plan for patients, such as whether to add neoadjuvant chemotherapy before surgery, and these aspects are usually evaluated by cystoscopy and imaging examination nowadays. Although these IFs are unlikely to replace cystoscopy and imaging examination in this regard, because they are easy to obtain, even if they are only of a limited diagnostic value, they could provide clinicians with more information to assist them in evaluating the condition of patients, so the research on the diagnostic value of IFs has a high clinical value. In addition, because the recurrence rate of bladder cancer after surgery is high [5], it is also meaningful to clarify the IFs in predicting the recurrence and survival of patients after total cystectomy. Although there are a few studies on the diagnostic and predictive values of IFs in patients undergoing total cystectomy [6–8], none of these studies have included all the IFs mentioned above, and no study has evaluated the diagnostic and predictive values of SII in these patients. Therefore, we conduct this study in the hope of providing more evidence in this field for future researchers and clinicians.

## 2. Patients and Methods

### 2.1. Study Subjects

This study was a retrospective study involving patients who were diagnosed with bladder cancer and underwent total cystectomy at Peking University Cancer Hospital from 2014 to 2020. Inclusion criteria included (a) adult patients aged 18 years or older with complete preoperative blood routine test results; (b) a preoperative diagnosis of bladder cancer with no previous treatment other than cystoscopy, TURBT, and neoadjuvant chemotherapy; and (c) underwent total cystectomy, confirmed by postoperative pathological reports as bladder urothelial carcinoma, and complete information on tumor grade and stage. Exclusion criteria included (a) patients with tumors in other sites; (b) patients with acute or chronic infectious diseases, or with immune system diseases; and (c) patients with myelosuppression after neoadjuvant chemotherapy, which might affect the blood routine test results.

### 2.2. Study Methods

For enrolled patients, the following information was collected through the electronic medical record system of the hospital: hospitalization number, age, sex, height, weight, smoking history, maximum diameter of tumor, tumor grade, tumor stage (TNM stage), leukocyte count, neutrophil count, monocyte count, lymphocyte count, and platelet count. All surgeries were performed by two doctors in Peking University Cancer Hospital according to the same surgical procedure, with the same extent of operation, so as to avoid surgical factors affecting the prognosis of patients. Pathological staging and histological grading of bladder cancer were based on the American Joint Commission TNM staging system on Bladder Cancer (seventh edition, 2010) [9]. All pathology reports were issued by the urology team in the Department of Pathology, Peking University Cancer Hospital. For patients with inconsistent pathology after total cystectomy compared with preoperative pathology, higher grading and staging results were used. All peripheral blood test values were the results of the patient's first fasting blood sampling after admission. NLR was calculated as neutrophil count/lymphocyte count, dNLR was calculated as neutrophil count/(leukocyte count − neutrophil count), PLR was calculated as platelet count/lymphocyte count, LMR was calculated as lymphocyte count/monocyte count, and SII was calculated as platelet count × neutrophil count/lymphocyte count.

### 2.3. Statistical Analyses

Descriptive statistics were used to summarize patient characteristics, categorical variables were presented as numbers and percentages, and continuous variables were presented as the mean and standard deviation. The *t*-test was used to compare continuous variables between high and low groups of IFs, and the chi-square test and cross-table test were used to compare categorical variables between these groups. Receiver operating characteristic (ROC) curves were drawn to calculate the area under curve (AUC) of IFs, and Youden index was calculated to select the best cut-off value. The Kaplan-Meier method and log-rank test were used to compare the differences of overall survival (OS) and recurrence-free survival (RFS) between groups with high and low levels of IFs. The Cox proportional hazards regression analysis was used to analyze whether IFs were independent risk factors for OS and RFS. ROC curves were plotted, and the best cut-off values were calculated using MedCalc version 19. The cut-off values of OS and RFS were calculated using X-tile version 3, and the remaining statistics were completed using Stata version 15. All tests were two-sided, and *P* < 0.05 was considered statistically significant.

## 3. Results

### 3.1. Patients' Characteristics

A total of 79 eligible patients were enrolled, including 70 (88.61%) males and 9 (11.39%) females, with an average age of 63.62 years. The proportion of smokers was 54.43%, and the average BMI was 25.63. Of these 79 patients, 4 (5.06%) had low-grade bladder cancer and 75 (94.94%) had high-grade disease. The vast majority of patients were diagnosed with muscle invasive disease, and 5 (6.33%) patients were diagnosed with Ta and 18 (22.78%) patients with T1 disease. All 23 patients had indications for total cystectomy according to EAU guideline because the tumor could not be completely resected by TURBT, uncontrolled hematuria, or imaging suggesting lymph node metastasis [4]. Approximately a quarter of patients had lymph node metastases, including 12 (15.19%) with N1 and 7 (8.86%) with N2. Four patients (5.06%) were clinically diagnosed with systemic metastasis and underwent total cystectomy due to uncontrollable hematuria. The mean values of leukocyte count, neutrophil count, lymphocyte count, platelet count, and monocyte count were within the normal range, and the corresponding mean values of NLR, dNLR, PLR, LMR, and SII were 3.22, 2.22, 168.97, 4.05, and 740.38, respectively. The patients' characteristics are shown in Table 1.

### 3.2. Diagnostic Value of IFs in Pathology

**Figure 1:**
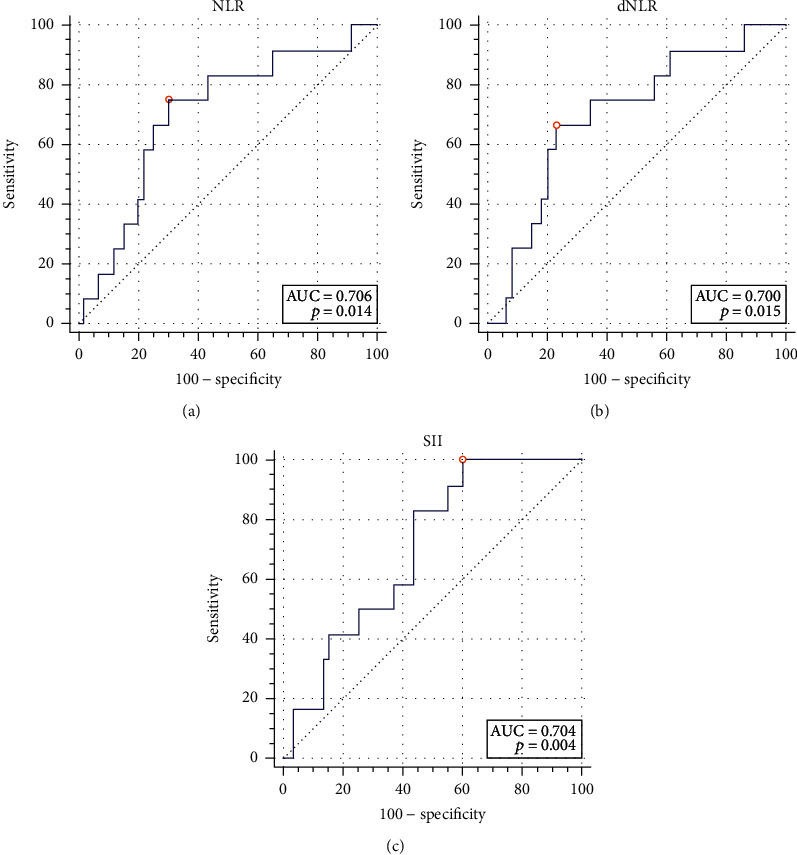
The receiver operating characteristic curves of NLR, dNLR, and SII for lymph node metastasis. (a) NLR had an acceptable diagnostic value for lymph node metastasis when the cut-off value was 3.12 (AUC = 0.706, sensitivity = 75.00%, specificity = 70.00%, *P* = 0.014). (b) dNLR had an acceptable diagnostic value for lymph node metastasis when the cut-off value was 2.49 (AUC = 0.700, sensitivity = 66.67%, specificity = 76.67%, *P* = 0.015). (c) SII had an acceptable diagnostic value for lymph node metastasis when the cut-off value was 463.56 (AUC = 0.704, sensitivity = 100.00%, specificity = 40.00%, *P* = 0.004).

The AUC of the diagnostic values of these IFs for pathological grade (low-grade, high-grade), tumor T stage (Ta-T4), lymph node metastasis (N0-N2), and systemic metastasis (M0, M1) are shown in Table 2. The results showed that among these IFs, all factors had no diagnostic value for the pathological grade, tumor T stage, and systemic metastasis of bladder cancer, and only NLR (AUC = 0.706, cut‐off value = 3.12, sensitivity = 75.00%, specificity = 70.00%, *P* = 0.014), dNLR (AUC = 0.700, cut‐off value = 2.49, sensitivity = 66.67%, specificity = 76.67%, *P* = 0.015), and SII (AUC = 0.704, cut‐off value = 463.56, sensitivity = 100.00%, specificity = 40.00%, *P* = 0.004) had a diagnostic value for lymph node metastasis. Among them, PLR and LMR had no diagnostic value for all items of pathological results. The relevant ROC curves are shown in Figure 1.

### 3.3. Association between Pathological Stage and Grade

Table 3 shows the association between pathological stage and grade. The results showed that the percentage of muscle invasion was significantly increased in high-grade tumors (*P* = 0.001), and the probability of lymph node metastasis was also significantly increased in muscle-invasive tumors (*P* = 0.009). The probability of muscle invasion was significantly increased in patients with larger tumor maximum diameter (*P* = 0.002). However, the probability of systemic metastasis was not significantly increased in patients with muscle invasion (*P* = 0.188).

### 3.4. Predictive Value of IFs in Survival and Recurrence

**Figure 2:**
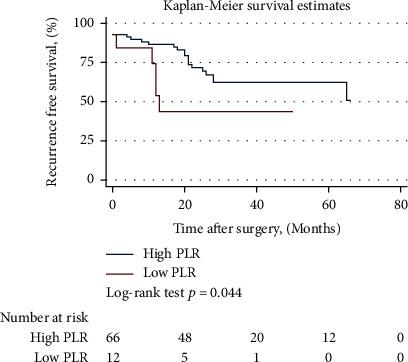
The Kaplan-Meier curve of RFS grouped by PLR. The low PLR group was significantly better than the high group (*P* = 0.044) when the cut-off value was 266.70, suggesting PLR has a predictive value for RFS.

With a median follow-up of 31 months, a total of 16 deaths and 22 recurrences were observed in 79 patients, of whom 9 died after recurrence. The best cut-off values of each inflammatory factor corresponding to OS and RFS calculated by X-tile software are shown in Table 4. All IFs were divided into high and low groups according to the cut-off value. The Kaplan-Meier curve analysis and log-rank test showed that there was no significant difference in OS between the two groups in these IFs. For RFS, the low PLR group was significantly better than the high group (*P* = 0.044) when the cut-off value was 266.70, suggesting that PLR has a predictive value for RFS (Figure 2). Considering the age of patients and TNM staging of tumors can affect the OS, the age and TNM staging were included in the Cox proportional hazards regression model for analysis, and the results showed that for OS, except for age (*P* = 0.005), all these IFs had no predictive value. For RFS, TNM staging of tumors was included in the Cox proportional hazards regression model for analysis because TNM staging of patients affected disease recurrence, and the results showed that all these IFs had no predictive value as well (Table 5).

## 4. Discussion

The blood routine test is a routine examination for all patients admitted to hospital. IFs can be obtained by simple calculation using existing items in the blood routine test. Because of the convenience, good repeatability, and low price, IFs have the potential to be widely used in clinic and have become a hot research field in recent years [2, 7]. NLR, dNLR, PLR, LMR, and SII are the IFs which have been more studied nowadays [6, 8], and the theory of these IFs predicting tumor pathology and prognosis is still not fully elucidated. Tachibana et al. found that bladder tumor cells can produce granulocyte colony-stimulating factor, which continuously stimulates the body to produce leukocytes [10]. Neutrophils can promote neocapillary formation by releasing elastase, breaking down tissue proteins, and destroying extracellular matrix, thus accelerating the growth and metastasis of tumor cells [11]. Platelets can affect the growth and metastatic dissemination of tumor cells; the main mechanism is that when tumor cells enter the blood system, they can induce platelets to aggregate around and wrap them, thus reducing the immunogenicity of tumor cells, assisting tumor cells to escape immune surveillance and clearance [12]. The decreasing of lymphocytes in tumor tissue leads to the weakening of the immunosuppressive effect of tumors [13]. The increasing of tumor-associated macrophages produced by monocytes is also related to the development of tumors [14]. Therefore, the IFs derived from the combination of the above two or three items have theoretical diagnostic and predictive values for tumors, and this value has been evaluated in gastric cancer, colorectal cancer, and breast cancer [15].

In terms of bladder cancer, many studies have found that IFs have a certain value in the diagnosis of bladder cancer [6]. Although the position of imaging and cystoscopy cannot be replaced by IFs, they can provide valuable reference for clinicians because of their simplicity. When clinicians find that patients' IFs are significantly higher, they should pay special attention to whether there is muscle invasion, lymph node metastasis, or systemic metastasis during cystoscopy and reading patients' images, which might affect the making of the treatment plan, so it is important to study the diagnostic value of IFs. According to relevant report, the diagnostic value of the AUC value between 0.7 and 0.8 is acceptable, the AUC value between 0.6 and 0.7 is poor, and the AUC value between 0.5 and 0.6 indicates no diagnostic value [16]. Our study found that all these IFs had no diagnostic value for pathological grade, tumor T stage, and systemic metastasis; only the AUC values of ROC curves of NLR, dNLR, and SII for lymph node metastasis were between 0.7 and 0.8, so they had an acceptable diagnostic value. Although the AUC value of PLR for the diagnosis of pathological grade was also between 0.7 and 0.8, but *P* > 0.05, PLR was also considered to be of no diagnostic value. It should be noted that the cut-off values of these IFs are different in different studies at present. In this study, for different items such as pathological grade and TNM stage of tumors, the optimal cut-off values are also different and even have large differences, such as both 1.80 and 3.12 are the cut-off values of NLR. Conclusions and the cut-off values are different in different research, which means that those studies are still at a very early stage, and there is still a large distance from the clinical application, which needs to be further clarified by future studies.

In terms of the correlation between pathological stage and grade, we found that high-grade tumors were more prone to muscle invasion, muscle invasion tumors were more prone to lymph node metastasis, and larger tumor maximum diameters were more prone to muscle invasion, which is consistent with our current clinical understanding.

Studies have suggested that IFs can predict the recurrence and survival of bladder cancer patients [17], but our study found that these IFs have no value in predicting the survival of patients, whether for OS or RFS. Although the Kaplan-Meier method found significant differences in RFS between low and high PLR groups, suggesting that PLR might have a predictive value for RFS, the subsequent Cox proportional hazards regression analysis showed that all these IFs had no predictive value for OS and RFS. One of the possible reasons that different studies have different results is the different sample size, and another reason might be that the cut-off values are different. It should be noted that in our study, the cut-off values for each inflammatory factor in OS and RFS are different, and the cut-off values used in different studies are also different, so it is still difficult to use these IFs in clinical practice to judge the condition and prognosis of patients. In addition, there are many factors affecting the survival of bladder cancer patients; in addition to age, pathological grade and stage, smoking, obesity, combination with basic diseases, postoperative adjuvant therapy, and so on might affect survival as well. Overall, there are many factors that need to be taken into account to predict the recurrence and survival of patients after surgery, which are relatively difficult. Combined with the findings of this study, we believe that these IFs have a limited value in this regard.

This study has some limitations; the main limitation is that it is a single-center study with small sample size and only 79 cases were enrolled; if the sample size can be further increased, there might be more positive findings. However, we used MedSci app on iOS to estimate the sample size. The expected sensitivity and specificity were set to 0.7, the allowable errors of sensitivity and specificity were set to 0.1, the test level (*α*) was set to 0.05, and the calculation result showed that the required sample size was 81 cases, which was close to the sample size of our study. We also searched for relevant studies and found the sample size was usually between 100 and 300 [18], but there were two similar studies in which 74 cases and 68 cases were enrolled, respectively [17, 19]. Considering the sample size estimated and relevant studies, we think the current sample size should also be acceptable. In turn, this single-center study brought about better consistency and controllability. The surgeries of all the patients in this study were performed by two urologists, which ensured the consistency of the operation. All pathological results were issued by the urology team in the Department of Pathology of our center, which also ensured the consistency of pathological evaluation criteria. Another limitation of this study is that the patient composition is relatively single. Since all the patients enrolled in this study were patients who underwent total cystectomy, the indications for surgery need to be met. In terms of tumor grade, only four patients were low-grade tumors, and the other 75 were high-grade tumors. Similarly, total cystectomy is not usually recommended in patients with distant metastases, so we have only four patients who underwent total cystectomy because of uncontrolled hematuria. The number of positive or negative groups is too small, which may affect the evaluation of the diagnostic value of the IFs. In terms of lymph node metastasis, positive results were obtained due to the relatively uniform distribution of patients with or without lymph node metastasis. Therefore, patient composition may also limit the analysis of the diagnostic value of these IFs. In addition, the median follow-up time in this study was 31 months, and a longer follow-up time might help to find more positive results for the predictive value of recurrence and survival. However, only one patient was followed up for less than half a year, six patients were followed up for less than one year, and 55 patients were followed up for more than two years. For these patients who underwent total cystectomy (70.89% of them were diagnosed with muscle invasive bladder cancer), we think this follow-up time is reasonable. The median follow-up time for similar studies is usually about 2-4 years [18].

Overall, we found that in patients undergoing total cystectomy preoperative NLR, dNLR and SII had a diagnostic value for lymph node metastasis, while all these five IFs had no predictive value for OS and RFS. However, because this study has some limitations, this conclusion needs to be further verified by large-scale studies in the future.

## Figures and Tables

**Table 1:** Patients' characteristics.

Characteristics	Total
Patients (*n*)	79
Age (year) (*x* ± *s*)	63.62 ± 7.52
Sex (*n*) (%)	
Male	70 (88.61)
Female	9 (11.39)
Smoking history (*n*) (%)	
Yes	43 (54.43)
No	36 (45.57)
BMI (*x* ± *s*)	25.63 ± 4.00
Maximum tumor diameter (cm) (*x* ± *s*)	3.80 ± 2.17
Grade (*n*) (%)	
Low grade	4 (5.06)
High grade	75 (94.94)
Tumor staging (*n*) (%)	
Ta	5 (6.33)
T1	18 (22.78)
T2	27 (34.18)
T3	26 (32.91)
T4	3 (3.80)
N0	60 (75.95)
N1	12 (15.19)
N2	7 (8.86)
M0	75 (94.94)
M1	4 (5.06)
Leukocyte count (×10^9^/L) (*x* ± *s*)	6.54 ± 1.92
Neutrophil count (×10^9^/L) (*x* ± *s*)	4.40 ± 1.72
Lymphocyte count (×10^9^/L) (*x* ± *s*)	1.56 ± 0.60
Platelet count (×10^9^/L) (*x* ± *s*)	232.95 ± 70.52
Monocyte count (×10^9^/L) (*x* ± *s*)	0.41 ± 0.15
NLR (*x* ± *s*)	3.22 ± 2.08
dNLR (*x* ± *s*)	2.22 ± 1.11
PLR (*x* ± *s*)	168.97 ± 80.33
LMR (*x* ± *s*)	4.05 ± 1.50
SII (*x* ± *s*)	740.38 ± 488.08

NLR: neutrophil to lymphocyte ratio; dNLR: derived neutrophil to lymphocyte ratio; PLR: platelet to lymphocyte ratio; LMR: lymphocyte to monocyte ratio; SII: systemic immune-inflammation index; BMI: body mass index.

**Table 2:** The AUC and cut-off values of the IFs for pathology.

	AUC	SE	95% CI		*Z* value	*P* value	Cut-off value	Sensitivity	Specificity
			LL	UL					
Grade									
NLR	0.523	0.145	0.408	0.637	0.160	0.873	2.71	50.67	25.00
dNLR	0.577	0.142	0.460	0.687	0.539	0.590	2.45	32.00	100.00
PLR	0.703	0.186	0.590	0.801	1.094	0.274	181.37	69.33	75.00
LMR	0.683	0.122	0.569	0.783	1.504	0.133	4.18	46.67	100.00
SII	0.547	0.135	0.431	0.659	0.347	0.729	650.98	60.00	75.00
T stage									
NLR	0.567	0.122	0.346	0.769	0.548	0.584	1.80	38.89	100.00
dNLR	0.511	0.135	0.297	0.723	0.082	0.935	2.45	66.67	0.00
PLR	0.667	0.138	0.442	0.847	1.208	0.227	172.82	77.78	60.00
LMR	0.644	0.113	0.420	0.830	1.282	0.200	4.63	50.00	100.00
SII	0.611	0.119	0.388	0.805	0.934	0.351	385.81	38.89	100.00
Lymph node metastasis									
NLR	0.706	0.083	0.586	0.807	2.464	0.014	3.12	75.00	70.00
dNLR	0.700	0.082	0.581	0.802	2.433	0.015	2.49	66.67	76.67
PLR	0.635	0.078	0.513	0.745	1.729	0.084	132.35	75.00	51.67
LMR	0.600	0.095	0.478	0.714	1.055	0.292	3.12	50.00	75.00
SII	0.704	0.071	0.585	0.806	2.890	0.004	463.56	100.00	40.00
Systemic metastasis									
NLR	0.625	0.094	0.509	0.731	1.335	0.182	3.00	100.00	41.33
dNLR	0.653	0.093	0.538	0.757	1.647	0.100	2.10	100.00	46.67
PLR	0.567	0.183	0.450	0.678	0.364	0.716	118.38	75.00	65.33
LMR	0.567	0.165	0.450	0.678	0.403	0.687	2.89	100.00	26.67
SII	0.547	0.152	0.431	0.659	0.307	0.759	511.91	75.00	60.00

NLR: neutrophil to lymphocyte ratio; dNLR: derived neutrophil to lymphocyte ratio; PLR: platelet to lymphocyte ratio; LMR: lymphocyte to monocyte ratio; SII: systemic immune-inflammation index; AUC: area under curve; 95% CI: 95% confidence interval; SE: standard error; IFs: inflammatory factors; LL: lower limit; UL: upper limit.

**Table 3:** The association between pathological stage and grade.

	Muscle invasion		Chi-square value	*P* value
	No	Yes		
Grade (*n*) (%)			10.259	0.001
Low grade	4 (100.00)	0 (0)		
High grade	19 (25.33)	56 (74.67)		
Lymph node metastasis (*n*) (%)			6.896	0.009
No	22 (36.67)	38 (63.33)		
Yes	1 (5.26)	18 (94.74)		
Systemic metastasis (*n*) (%)			1.731	0.188
No	23 (30.67)	52 (69.33)		
Yes	0 (0.00)	4 (100.00)		
Maximum tumor diameter (*n*) (%)			9.168	0.002
<3 cm	14 (50)	14 (50)		
≥3 cm	9 (17.65)	42 (82.35)		

**Table 4:** The Kaplan-Meier results of OS and RFS grouped by the IFs.

	Cut-off value	Low group		High group		*P* value
		Events observed	Events expected	Events observed	Events expected	
OS						
NLR	2.00	5	4.18	11	11.82	0.638
dNLR	1.80	10	7.35	6	8.65	0.182
PLR	133.30	10	7.13	6	8.87	0.148
LMR	2.80	6	3.28	10	12.72	0.090
SII	547.30	10	7.74	6	8.26	0.258
RFS						
NLR	3.10	12	14.52	10	7.48	0.254
dNLR	1.50	5	6.92	17	15.08	0.375
PLR	266.70	17	19.80	5	2.20	0.044
LMR	4.70	14	15.85	8	6.15	0.371
SII	778.00	13	15.72	9	6.28	0.195

NLR: neutrophil to lymphocyte ratio; dNLR: derived neutrophil to lymphocyte ratio; PLR: platelet to lymphocyte ratio; LMR: lymphocyte to monocyte ratio; SII: systemic immune-inflammation index; OS: overall survival; RFS: recurrence-free survival; IFs: inflammatory factors.

**Table 5:** The Cox proportional hazards regression of OS and RFS.

	HR	SE	95% CI		*Z* value	*P* value
			LL	UL		
OS						
NLR	1.319	0.986	0.305	5.711	0.370	0.711
dNLR	0.615	0.718	0.063	6.052	-0.420	0.677
PLR	0.993	0.007	0.979	1.008	-0.880	0.376
LMR	1.125	0.330	0.633	1.998	0.400	0.689
SII	1.000	0.002	0.996	1.004	0.040	0.967
Age	1.098	0.037	1.028	1.172	2.780	0.005
T stage	2.144	0.931	0.915	5.023	1.760	0.079
N stage	2.238	0.967	0.960	5.217	1.860	0.062
M stage	3.815	3.314	0.695	20.942	1.540	0.123
RFS						
NLR	0.537	0.460	0.100	2.882	-0.720	0.469
dNLR	1.149	1.267	0.132	9.975	0.130	0.899
PLR	1.004	0.004	0.995	1.012	0.890	0.375
LMR	1.098	0.234	0.724	1.666	0.440	0.659
SII	1.001	0.002	0.998	1.004	0.620	0.534
T stage	1.804	0.595	0.946	3.442	1.790	0.073
N stage	1.977	0.730	0.958	4.079	1.840	0.065
M stage	2.458	2.021	0.490	12.321	1.090	0.274

NLR: neutrophil to lymphocyte ratio; dNLR: derived neutrophil to lymphocyte ratio; PLR: platelet to lymphocyte ratio; LMR: lymphocyte to monocyte ratio; SII: systemic immune-inflammation index; OS: overall survival; RFS: recurrence-free survival; HR: hazard ratio; SE: standard error; 95% CI: 95% confidence interval; LL: lower limit; UL: upper limit.

## Data Availability

All authors had full access to all the data in the study and take responsibility for the integrity of the data and the accuracy of the data analysis. The datasets generated and/or analyzed during the current study are available from the corresponding author on reasonable request.
